# Theoretical investigations on mechanisms and kinetics of the CH_3_CFClO_2_· with ClO· reaction in the atmosphere

**DOI:** 10.1038/s41598-020-68049-4

**Published:** 2020-07-06

**Authors:** Yunju Zhang, Bing He, Yuxi Sun

**Affiliations:** 1 Key Laboratory of Photoinduced Functional Materials, Mianyang Normal University, Mianyang, 621000 People’s Republic of China; 2 College of Chemistry and Life Science, Institute of Functional Molecules, Chengdu Normal University, Chengdu, 611130 Sichuan People’s Republic of China

**Keywords:** Computational science, Theoretical chemistry, Computational chemistry, Reaction mechanisms, Mathematics and computing, Environmental sciences, Environmental chemistry

## Abstract

The singlet and triplet potential energy surfaces of the ClO• radical reaction with the CH_3_CFClO_2_• radical have been investigated at the CCSD(T)/cc-pVTZ level based on the optimized geometries at the B3LYP/6–311++G(d,p) level. On the singlet potential energy surfaces (PES), the possible reaction involves association-dissociation, direct H-abstraction and Nucleophilic Substitution 2 (S_N_2) mechanisms. On the triplet PES, S_N_2 displacement and direct H-abstraction reaction pathways have been investigated, which are less competitive compared with the reaction pathways on the singlet PES. The rate constants have been calculated at 10^–10^ to 10^10^ atm and 200–3,000 K by Rice–Ramsperger–Kassel–Marcus (RRKM) theory for the important product pathways. At 200–800 K, IM1 produced (CH_3_CFClOOOCl) by collisonal deactivation is dominant; at high temperatures, the production P1 (CH_3_CFO + ClOOCl) becomes dominate. The calculated rate constants for CH_3_CFClO_2_• + ClO• are good agreement with the available experimental value. The atmospheric lifetime of CH_3_CFClO_2_• in ClO• is around 3.27 h. TD-DFT computations imply that IM1 (CH_3_CFClOOOCl), IM2 (CH_3_CFClOOClO) and IM3 (CH_3_CFClOClO_2_) will photolyze under the sunlight.

## Introduction

ClO• radical is an active halogen species, and it is abundant in the atmosphere. It is of great atmospheric significance due to its ability to destroy ozone. ClO• plays a major role in the formation of the Antarctic “ozone hole”, through the following catalytic cycle^1^:

$$ \begin{gathered} {\text{ClO + ClO + M}} \to {\text{ClOOCl } + \text{ M}} \hfill \\ {\text{ClOOCl}} + hv \to {\text{Cl } + \text{ ClOO}} \hfill \\ {\text{ClOO } + \text{ M}} \to {\text{Cl}} + {\text{O}}_{2} + {\text{M}} \hfill \\ {\text{2*(Cl + O}}_{3} \to {\text{ClO } + \text{ O}}_{{2}} ) \hfill \\ {\text{Net:2O}}_{3} + hv \to 3{\text{O}}_{2} \hfill \\ \end{gathered} $$

The production and photolysis for the dipolymer of ClO• (ClOOCl) are necessary for the chemistry in the above process. According to statistics, this cycle brought about 70% destruction of the Antarctic ozone^2^. However, recent studies have suggested that this gas-phase chemistry alone cannot account for ozone loss due to chlorine-catalyzed loss of ozone^3^.

The CH_3_CFClO_2_• radical is predicted to be produced by the photolysis of the CH_3_CFCl_2_ in the presence of excessive oxygen^4^

$$ \begin{gathered} {\text{CH}}_{3} {\text{CFCl}}_{2} + hv \to {\text{CH}}_{3} {\text{CFCl}} + {\text{Cl}} \hfill \\ {\text{CH}}_{3} {\text{CFCl}} + {\text{O}}_{2} \to {\text{CH}}_{3} {\text{CFClO}}_{2} \hfill \\ \end{gathered} $$

The possible degradation mechanism of the peroxy radicals includes self-reactions and reactions with radicals, i.e. •Cl, ClO• and NO^4,5^. In particular, the reaction of alkyl peroxide radical with ClO• is an interesting system, which has an important influence on stratospheric ozone chemistry and CH_3_CFCl_2_ oxidation chain. Previous research^5^ have estimated that the dominant products of the CH_3_CFClO_2_• + ClO• reaction are alkoxy chloride (CH_3_CFClOCl), oxygen molecule, alkoxy group (CH_3_CFClO) and chlorine peroxide (ClOO).

$$ \begin{gathered} {\text{CH}}_{3} {\text{CFClO}}_{2} + {\text{ClO}} \to {\text{CH}}_{3} {\text{CFClOCl}} + {\text{O}}_{2} \hfill \\ {\text{CH}}_{3} {\text{CFClO}}_{2} + {\text{ClO}} \to {\text{CH}}_{3} {\text{CFClO } + \text{ ClOO}} \hfill \\ \end{gathered} $$

Wu and Carr^5^ investigated the with CH_3_CFClO_2_• + ClO• reaction by UV flash photolysis and time-resolved mass spectrometry at 253–321 K and 4–60 Torr, and the rate constants were estimated to be (4.5 ± 1) × 10^–12^ cm^3^molecule^-1^ s^-1^ in the temperature range of these experiments. In this work, a detailed mechanism investigation of the CH_3_CFClO_2_• + ClO• reaction was performed by means of quantum chemical calculations. The rate constants of the dominant reaction pathways of the CH_3_CFClO_2_• + ClO• reaction were calculated by RRKM theory^6^, which has been employed to deal with the complex reactions successfully^7–12^.

### Computational methods

All calculations in the present study were preformed using the GAUSSIAN09 program^13^. The geometries of some important intermediates and transition states (IM1, IM2, IM3, TS1, TS2, TS3, TS7 and TS10) were optimized with density functional theory (B3LYP^14,15^ and M06-2X^16,17^ functionals) with the same triple-§ 6–311++G(d,p) basis set. The other geometries on potential energy surfaces were calculated using the B3LYP/6–311++G(d,p) method. All stationary points were characterized by harmonic vibrational frequency analysis (the number of imaginary frequencies, NIMAG, 0 for minima and 1 for transition states). In addition, we calculated the intrinsic reaction coordinates (IRC)^18,19^ to verify the connectivity between transition state and the corresponding reactants or products. Based on the B3LYP optimized geometry structures, the time-dependent functional theory (TDDFT) theory with the DFT/B3LYP methods with 6–311++G(d,p) as basis set was used to obtain the vertical excitation energy (*T*_V_) of all the intermediates in the CH_3_CFClO_2_• + ClO• reaction. In order to obtain more reliable relative energy, single points energy calculations have been performed by the method of CCSD(T)^20^/cc-pVTZ using the functional B3LYP-D3(BJ).

## Results and discussion

**Figure 1:**
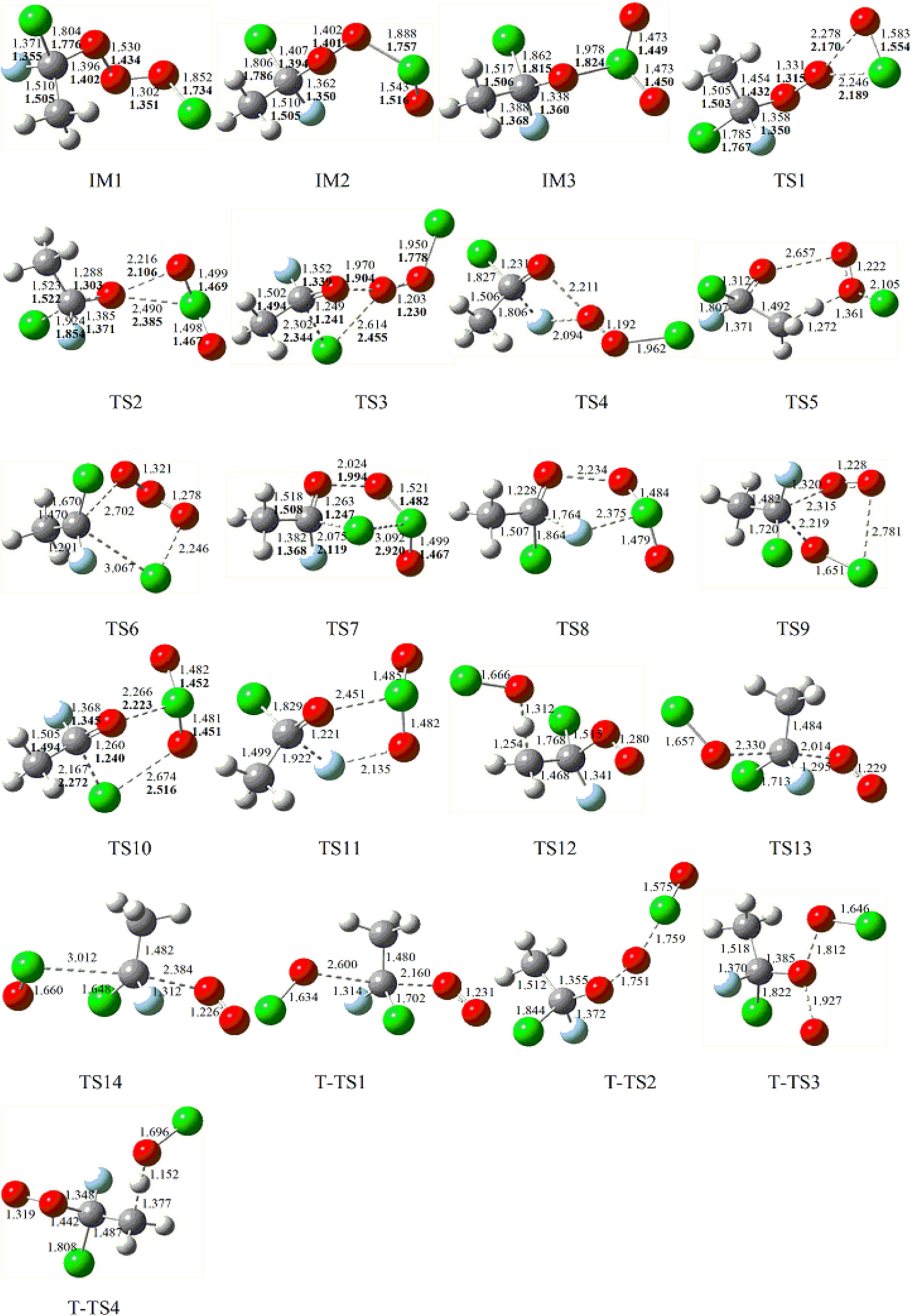
CH_3_CFClO_2_• with ClO• reaction: optimized B3LYP geometries of the intermediates and transition stats at B3LYP/6–311 +  + G(d,p) level. The values in bold are obtained at M06-2X/6–311 +  + G(d,p) level. Bond distances are given in Å.

**Figure 2:**
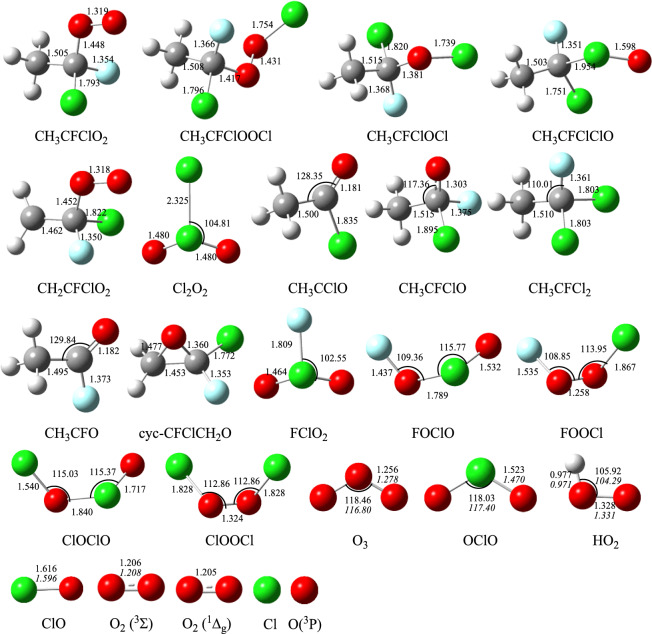
CH_3_CFClO_2_• with ClO• reaction: optimized B3LYP geometries of the reactants and products. Angles are given in º, and bond distances are given in Å. The values in italics are experimental data from ref 21.

**Figure 3:**
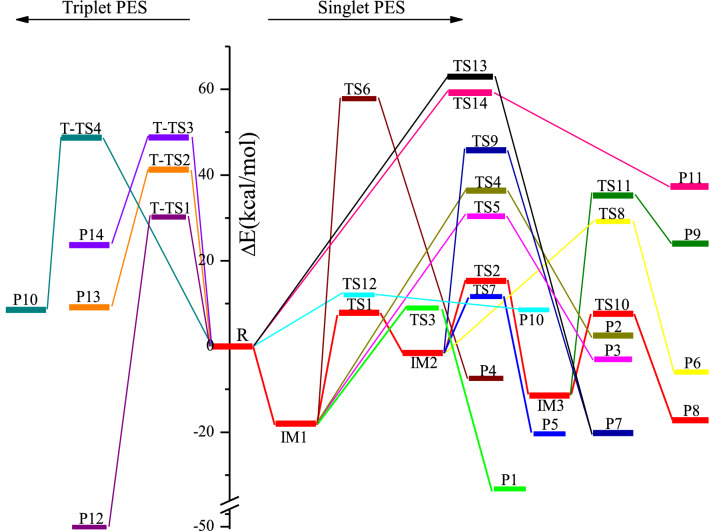
Potential energy surface obtained at CCSD(T)//B3LYP level for the CH_3_CFClO_2_• + ClO• reaction.

The optimized geometries of the intermediates and transition stats involved on the triplet and singlet PESs in the title reaction at the B3LYP/6–311++G(d,p) level are depicted in Fig. 1. The optimized geometries for the reactants and products are shown in Fig. 2, along with the available experimental values^21^. All possible pathways involved in the CH_3_CFClO_2_• + ClO• reaction are presented in Fig. 3. Table 1 summarizes ZPE corrections, relative energies, reaction enthalpies and Gibbs free energy. The harmonic vibrational frequencies, moment of inertia and the Z-matrix Cartesian coordinate of all species found on the PESs as supplementary materials are shown in Tables S1, S2, respectively. The frequencies of CH_3_CFO, CH_3_CClO, OClO, HOCl, ClO•, HO_2_, O_3_ and O_2_(^3^Σ) are in agreement with experimental data^21^. The energies obtained at CCSD(T)/cc-pVTZ//B3LYP/6–311++G(d,p) level are employed to the following discussion.

**Table 1:** Zero Point Energies (ZPE) and relative Energies (ΔE), relative enthalpies (ΔH) and Gibbs free energy (ΔG) for the species involved in the CH_3_FClO_2_• with ClO• reaction (energies in kcal/mol).

**Species**	**ZPE**^**a**^	**ΔE**^**b**^	**ΔH**^**b**^	**ΔG**^**b**^
R: (CH_3_CFClO_2_• + ClO•)	34.70	0.00	0.00	0.00
IM1: (CH_3_CFClOOOCl)	36.40	− 18.01	− 18.44	− 7.21
IM2: (CH_3_CFClOOClO)	36.09	− 1.53	− 1.76	9.09
IM3: (CH_3_CFClOClO_2_)	36.62	− 11.42	− 11.76	− 0.85
TS1	35.89	7.84	7.23	18.64
TS2	35.26	15.33	15.04	25.46
TS3	35.33	8.96	8.85	19.06
TS4	34.54	36.37	36.48	46.10
TS5	32.20	30.39	30.06	40.49
TS6	34.87	57.80	57.72	67.95
TS7	35.15	11.64	11.34	22.45
TS8	34.72	29.18	29.08	39.72
TS9	34.79	45.76	45.63	56.43
TS10	35.56	7.63	7.31	18.29
TS11	34.69	35.25	35.26	45.09
TS12	31.53	12.05	11.94	21.49
TS13	34.18	62.97	63.33	72.62
TS14	33.59	59.24	60.01	67.84
T-TS1	33.43	30.18	30.94	37.89
T-TS2	33.96	41.25	41.35	50.09
T-TS3	33.74	48.80	48.97	57.73
T-TS4	31.04	48.72	48.67	57.48
P1: (CH_3_CFO + ClOOCl)	34.64	− 33.25	− 33.15	− 34.94
P2: (CH_3_CClO + FOOCl)	34.29	2.55	2.68	0.86
P3: (cyc-CFClCH_2_O + HO_2_ + Cl)	33.93	− 2.97	− 2.41	− 11.14
P4: (CH_3_CFCl_2_ + O_3_)	35.05	− 7.46	− 7.67	− 7.34
P5: (CH_3_CFO + Cl_2_O_2_)	35.36	− 20.36	− 20.37	− 21.81
P6: (CH_3_CClO + FClO_2_)	35.06	− 5.98	− 6.04	− 7.26
P7: (CH_3_CFClOCl + O_2_(^1^Δ_g_))	35.12	− 20.17	− 20.07	− 18.49
P8: (CH_3_CFO + ClOClO)	34.20	− 17.20	− 16.86	− 19.17
P9: (CH_3_CClO + FOClO)	33.73	23.98	24.32	22.15
P10: (CH_2_CFClO_2_ + HOCl)	32.43	8.53	9.04	7.38
P11: (CH_3_CFClClO + O_2_(^1^∆_g_))	33.83	37.31	37.85	38.15
P12: (CH_3_CFClOCl + O_2_(^3^∑))	35.14	− 50.32	− 50.23	− 49.30
P13: (CH_3_CFClO + OClO)	33.82	9.14	9.17	7.50
P14: (CH_3_CFClOOCl + O(^3^P))	34.57	23.68	23.96	26.42

^a^At the B3LYP/6–311 +  + G(d,p) level.

^b^The relative energies are calculated at the CCSD(T)//B3LYP/6–311 +  + G(d,p) level.

### The formation of adducts on the singlet PES

The ClO• + CH_3_CFClO_2_• reaction initiated by the oxygen or chlorine atom of ClO• radical addition to the terminal-O atom of CH_3_CFClO_2_• without a barrier and produced the original adduct IM1 (CH_3_CFClOOOCl) or IM2 (CH_3_CFClOOClO) with exothermicity of 18.44 kcal/mol or 1.76 kcal/mol. The formed O–O and O**–**Cl bonds are 1.302 and 1.888 Å in IM1 and IM2, respectively. IM1 could isomerize to IM2 via a ClOO triangular structure TS1, in which the O–Cl bond that will be formed is 2.246 Å, while the distance of breaking O–O bond is 2.278 Å. TS1 lies 26.28 and 7.84 kcal/mol above IM1 and reactants, respectively. In addition, IM2 can isomerize to IM3 (CH_3_CFClOClO_2_) via the transition state TS2 with a barrier of 16.86 kcal/mol, in whch the –ClO group insert into the O–O bond between the terminal and the middle O atoms, and the O–O bond breakage simultaneously. The breaking O–O bond in the triangular structure TS2 is stretched to 2.216 Å and the forming O–Cl bond is 2.490 Å. The imaginary frequency of TS2 is 157*i* cm^-1^, involving the O–O and O–Cl bonds stretch vibrations simultaneously. In a word, three adducts IM1 (CH_3_CFClOOOCl), IM2 (CH_3_CFClOOClO) and IM3 (CH_3_CFClOClO_2_) are generated on the singlet PES with the energy of − 18.01, − 1.53 and − 11.42 kcal/mol, which could further dissociate to various products, and will be discussed as bellow.

### The decomposition pathways from IM1 (CH_3_CFClOOOCl), IM2 (CH_3_CFClOOClO) and IM3 (CH_3_CFClOClO_2_)

Starting from IM1, the O–O bond decomposes and the Cl or F atom in –CFCl– group moves to the middle-O atom in the –OOO– skeleton synchronously resulting in P1 (CH_3_CFO + ClOOCl) or P2 (CH_3_CClO + FOOCl) via a ClCOO or FCOO four-numbered-ring transition state TS3 or TS4. The broken O–O and C–Cl bonds are about 1.970 and 2.302 Å in TS3, and the broken O–O and C-F bonds are 2.211 and 1.806 Å in TS4. The produced Cl–O bond in TS3 and F–O bond in TS4 are 2.614 and 2.094 Å, respectively. The barriers for these two decomposition channels are 26.97 and 54.38 kcal/mol, respectively. The IM1 → TS3 → P1 pathway is exothermic by 33.15 kcal/mol, and the IM1 → TS4 → P2 pathway is endothermic by 2.68 kcal/mol. The overall ΔG for these two decomposition channels are − 34.94 and 0.86 kcal/mol, indicating that the pathway via TS3 is practicable thermodynamically.

1,5-H migration from the –CH_3_ group to the middle-O atom in the –OOCl skeleton, associated with the O–O and O–Cl bonds fracturing through TS5 gives rise to P3 (cyc-CFClCH_2_O + HO_2_ + Cl). In TS5, the C–H bond (1.272 Å), O–O bond (2.657 Å) and the C–Cl bond (2.105 Å) are elongated by 0.183, 1.127 and 0.253 Å compared with those of in IM1 (1.089, 1.530 and 1.852 Å, respectively), and the formed O–H bond is 1.361 Å. Although the overall exothermicities of forming P3 (cyc-CFClCH_2_O + HO_2_ + •Cl) pathway is estimated to be 2.41 kcal/mol, the barrier for IM1 → TS5 → P3 is 48.40 kcal/mol. To any extent, the high barrier restrains the dissociation pathways from proceeding.

Besides the above three decomposition pathways from IM1 (CH_3_CFClOOOCl), the reaction resulting in P4 (CH_3_CFCl_2_ + O_3_) takes place by synchronously the migration of the terminal Cl atom to the carbon atom of –CFCl– group and breaking of the C–O bond through a COOOCl five-center structure TS6. The activation barrier of the IM1 → TS6 → P4 process is 75.81 kcal/mol. Apparently, this decomposition channel is not important to the overall reaction.

IM2 (CH_3_CFClOOClO) could take place decomposition into the end product P5 (CH_3_CFO + Cl_2_O_2_) or P6 (CH_3_CClO + FClO_2_) through five-center structure TS7 or TS8, respectively. These two decomposition channels involve the Cl atom or F atom of the –CFCl– group migrating to the chlorine atom of the –OOClO skeleton, accompanied by the O–O bond splitting, respectively. In TS7, the C–Cl bond (2.075 Å) and the O–O bond (2.024 Å) are elongated by 0.269 and 0.622 Å compared with the corresponding bond in IM2 (1.806 and 1.402 Å, respectively), and the formed Cl-Cl bond is 3.092 Å. In TS8, the C-F bond (1.764 Å) and the O–O bond (2.234 Å) are elongated by 0.402 and 0.832 Å, and the formed F-Cl bond is 2.375 Å. Vibrational frequency analysis of TS7 or TS8 reveal one imaginary frequency of 242*i* and 469*i* cm^−1^. The activation barriers for IM2 → TS7 → P5 and IM2 → TS8 → P6 are 13.17 and 30.71 kcal/mol, and the relative energy of P5 and P6 are − 20.36 and − 5.98 kcal/mol. Therefore, the pathway of leading to P5 (CH_3_CFO + Cl_2_O_2_) has priority over the formation of P6 (CH_3_CClO + FClO_2_) pathway.

Additionally, IM2 (CH_3_CFClOOClO) could decompose to P7 (CH_3_CFClOCl + O_2_ (^1^Δ_g_)) via COOClO five-center structure TS9 with the barrier of 47.29 kcal/mol. In TS9, the terminal-O atom shifts to the carbon atom of the –CFCl- group, associated with the breakage of C–O and Cl–O bonds. Though the Δ*H* and Δ*G* for this channel are − 20.07 and − 18.49 kcal/mol, this dissociation pathway is unfavorable due to the high barrier height.

IM3 (CH_3_CFClOClO_2_) could involve 1,4-Cl shift and O-Cl bond breaking producing P8 (CH_3_CFO + ClOClO) via TS10, or may take 1,4-F-shift and O-Cl bond breaking resulting in P9 (CH_3_CClO + FOClO) via TS11. The decomposition barriers for IM3 → TS10 → P8 and IM3 → TS11 → P9 are 19.05 and 46.67 kcal/mol, respectively. The loose ClCOClO and FCOClO five-membered ring in TS10 and TS11 are nonplanar. The migrating chlorine is away from the origin-C atom of 2.167 Å, and 2.674 Å away from the shifting terminus oxygen atom, and the O–Cl bond that will be disruption is as long as 2.266 Å. The vibrational mode of frequency of TS11 corresponds to O–Cl, C–F and O–F bonds stretch vibration, that is, *r*(O–Cl) = 2.451 Å, *r*(C–F) = 1.922 Å, and *r*(O–F) = 2.135 Å. The enthalpy of P8 and P9 are − 17.20 and 23.98 kcal/mol and the Gibbs free energy of P8 and P9 are − 19.17 and 22.15 kcal/mol, imply that IM3 → TS10 → P8 is exothermic and spontaneous, and IM3 → TS11 → P9 is endothermic and nonspontaneous.

### Direct H-abstraction pathways on the singlet PES

One direct H-abstraction pathway is found for the CH_3_CFClO_2_• + ClO• reaction. One of the H atoms in CH_3_CFClO_2_• is abstracted by the O atom in ClO• via TS12 to form P10 (CH_2_CFClO_2_ + HOCl). The distance of the breaking C–H bond is 1,254 Å, and forming O–H bond is 1.312 Å. We can define a parameter which represents the reactant- or product-like character of the forming transition state. The L parameter could be computed with the expression^22–25^: $L(C - {\text{H}}) = \frac{{\delta r\left( {{\text{C}} - {\text{H}}} \right)}}{{\delta r\left( {{\text{O}} - {\text{H}}} \right)}}$, where $\delta r\left( {{\text{C}} - {\text{H}}} \right)$ and $\delta r\left( {{\text{O}} - {\text{H}}} \right)$ are the corresponding bond distance variations between the TS12 structure and the reactant CH_3_CFClO_2_• for the C–H bond and between the TS12 structure and the product HOCl for the O–H bond. The L parameter denotes if a transition state structure is reactant-like (L < 1) or product-like (L > 1) and also quantifies the corresponding trend. The value of L parameter for TS12 is 0.47, indicating that TS12 is a reactant-like transition state. The barrier for CH_3_CFClO_2_• + ClO• → TS12 → P10 (CH_2_CFClO_2_ + HOCl) pathway is 12.05 kcal/mol, which may be important for the reaction.

### The S_N_2 displacement pathways on the singlet PES

Beside the above addition/elimination and direct H-abstraction pathways, S_N_2 displacement reaction could occur through TS13 or TS14 with the O atom or the Cl atom of ClO• attacking the carbon atom of the –CFCl– group in CH_3_CFClO_2_•, accompanied by the O_2_ (^1^Δ_g_) leaving away. In TS13 and TS14, the distance of the forming C–O and C–Cl bonds are 2.330 and 3.012 Å, and the breaking C–O bonds are stretched to 2.014 and 2.384 Å, respectively. The barriers for these two S_N_2 displacement reactions are rather high, 62.97 and 59.24 kcal/mol, making they are unimportant and could be excluded.

### The pathways on the triplet PES

Many attempts failed to locate the intermediate on the triplet PES. Based on our results, three S_N_2 displacement and one direct H-abstraction channels were found. Figure 1 displays that surmounting T-TS1, T-TS2, T-TS3 and T-TS4, P12 (CH_3_CFClOCl + O_2_ (^3^∑)), P13 (CH_3_CFClO + OClO), P14 (CH_3_CFClOOCl + O(^3^P)) and P10 (CH_2_CFClO_2_ + HOCl) are produced, and the corresponding relative energy of the products is − 50.32, 9.14, 23.68 and 8.53 kcal/mol, respectively. The relative energy of T-TS1, T-TS2, T-TS3 and T-TS4, are 30.18, 41.25, 48.80 and 48.72 kcal/mol. It is suggested that the direct H-abstraction and all S_N_2 displacement channels on the triplet PES contribute less to the title reaction judging from the high barriers.

### Kinetics

**Scheme 1:**
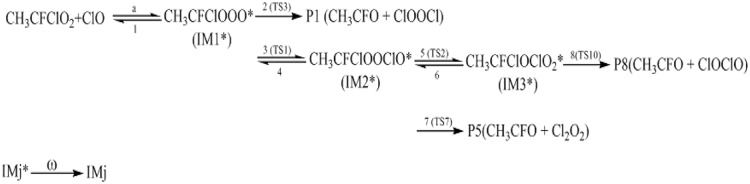
The primary reaction pathways for the CH_3_CFClO_2_• with ClO• reaction.

As discussed above, for the reaction pathways producing P1, P5 and P8 (Scheme 1), the reaction energy barriers are lower and the reactions are exothermic, so these reaction pathways are involved in the kinetics calculations. However, the reaction pathways producing P2, P3, P4, P5, P7, P9, P10, P11, P12, P13 and P14 with higher energy barrier are less competitive in energy, and their contribution to the total reaction is negligible.

Temperature- or pressure-dependent rate constants for the important pathways (Scheme 1) of the CH_3_CFClO_2_• + ClO• reaction were computed at 200–3,000 K, 10^–10^–10^10^ atm utilizing RRKM theory. The kinetics calculations based on the optimized geometries, moment of inertia and frequencies obtained at B3LYP/6–311 +  + G(d,p) level, and the energies obtained at the CCSD(T)/cc-pVTZ level.

Steady-state assumption for all the excited (IMj) generates to the following expressions: for the second-order rate constants of diverse product pathways:

1 $$ k_{{\rm{IM1}}} (T,P) = \frac{{\alpha_{a} }}{h}\frac{{Q_{t}^{ \ne } Q_{r}^{ \ne } }}{{Q_{{\rm{CH}_{3} \rm{CFClO}_{\rm{2}} \bullet }} Q_{{\rm{ClO} \bullet_{{}} }} }}e^{{ - E_{a} /RT}} \times \int_{0}^{\infty } {\frac{\omega }{{X_{5} }}} N_{a} (E^{ \ne } )e^{{ - E^{ \ne } /RT}} dE^{ \ne } $$

2 $$ k_{{\rm{IM2}}} (T,P) = \frac{{\alpha_{a} }}{h}\frac{{Q_{t}^{ \ne } Q_{r}^{ \ne } }}{{Q_{{\rm{CH}_{3} \rm{CFClO}_{\rm{2}} \bullet }} Q_{{\rm{ClO} \bullet_{{}} }} }}e^{{ - E_{a} /RT}} \times \int_{0}^{\infty } {\frac{{\omega X_{3} }}{{X_{5} }}} N_{a} (E^{ \ne } )e^{{ - E^{ \ne } /RT}} dE^{ \ne } $$

3 $$ k_{{\rm{IM3}}} (T,P) = \frac{{\alpha_{a} }}{h}\frac{{Q_{t}^{ \ne } Q_{r}^{ \ne } }}{{Q_{{\rm{CH}_{3} \rm{CFClO}_{\rm{2}} \bullet }} Q_{{\rm{ClO} \bullet_{{}} }} }}e^{{ - E_{a} /RT}} \times \int_{0}^{\infty } {\frac{{\omega X_{{1}} X_{3} }}{{X_{5} }}} N_{a} (E^{ \ne } )e^{{ - E^{ \ne } /RT}} dE^{ \ne } $$

4 $$ k_{{\rm{P1}}} (T,P) = \frac{{\alpha_{a} }}{h}\frac{{Q_{t}^{ \ne } Q_{r}^{ \ne } }}{{Q_{{\rm{CH}_{3} \rm{CFClO}_{\rm{2}} \bullet }} Q_{{\rm{ClO} \bullet_{{}} }} }}e^{{ - E_{a} /RT}} \times \int_{0}^{\infty } {\frac{{k_{2} (E)}}{{X_{5} }}} N_{a} (E^{ \ne } )e^{{ - E^{ \ne } /RT}} dE^{ \ne } $$

5 $$ k_{{\rm{P2}}} (T,P) = \frac{{\alpha_{a} }}{h}\frac{{Q_{t}^{ \ne } Q_{r}^{ \ne } }}{{Q_{{\rm{CH}_{3} \rm{CFClO}_{\rm{2}} \bullet }} Q_{{\rm{ClO} \bullet_{{}} }} }}e^{{ - E_{a} /RT}} \times \int_{0}^{\infty } {\frac{{k_{7} (E)X_{{3}} }}{{X_{5} }}} N_{a} (E^{ \ne } )e^{{ - E^{ \ne } /RT}} dE^{ \ne } $$

6 $$ k_{{\rm{P8}}} (T,P) = \frac{{\alpha_{a} }}{h}\frac{{Q_{t}^{ \ne } Q_{r}^{ \ne } }}{{Q_{{\rm{CH}_{3} \rm{CFClO}_{\rm{2}} \bullet }} Q_{{\rm{ClO} \bullet_{{}} }} }}e^{{ - E_{a} /RT}} \times \int_{0}^{\infty } {\frac{{k_{8} (E)X_{1} X_{3} }}{{X_{5} }}} N_{a} (E^{ \ne } )e^{{ - E^{ \ne } /RT}} dE^{ \ne } $$

With the following definition:

$$ \begin{gathered} {\text{X}}_{{1}} = k_{{5}} \left( E \right)/(k_{{6}} \left( E \right) + k_{{8}} \left( E \right) + \omega ) \hfill \\ {\text{X}}_{{2}} = k_{{4}} \left( E \right) + k_{{5}} \left( E \right) + k_{{7}} \left( E \right) + \omega \hfill \\ {\text{X}}_{{3}} = k_{{3}} \left( E \right)/({\text{X}}_{{2}} - k_{{5}} \left( E \right)*{\text{X}}_{{1}} ) \hfill \\ {\text{X}}_{{4}} = k_{{1}} \left( E \right) + k_{{2}} \left( E \right) + k_{{3}} \left( E \right) + \omega \hfill \\ {\text{X}}_{{5}} = {\text{X}}_{{4}} - k_{{4}} \left( E \right)*{\text{X}}_{{3}} \hfill \\ \end{gathered} $$

The microcanonical rate constant is calculated using the RRKM theory as follows:

7 $$ k_{i} (E) = \alpha_{i} C_{i} N_{i} (E_{i}^{ \ne } )/h\rho_{j} (E_{j} ) $$

In the above equations, $\alpha_{\alpha }$ is the statistical factor for the reaction path a, and α_*i*_ is the statistical factor (degeneracy) for the *i*th reaction path; *E*_a_ is the energy barrier for the reaction step a. $Q_{{\rm{ClO} \bullet }}$ and $Q_{{\rm{CH}_{3} \rm{CFClO}_{\rm{2}} \bullet }}$ are the total partition function of ClO• and CH_3_CFClO_2_•, respectively; $Q_{t}^{ \ne }$ and $Q_{r}^{ \ne }$ are the translational and rotational partition functions of entrance transition state, respectively; $N_{a} \left( {E^{ \ne } } \right)$ is the number of state for the association transition state with excess energy $E^{ \ne }$ above the association barrier. $k_{i} \left( E \right)$ is the energy-specific rate constant for the *i*th channel and C_*i*_ is the ratio of the overall rotational partition function of the TS_*i*_ and IM_*j*_; $N_{i} \left( {E_{i}^{ \ne } } \right)$ is the number of states at the energy above the barrier height for transition state *i*; $\rho_{j} \left( {E_{j} } \right)$ is the density of states at energy *E*_*j*_ of the intermediate. The density of states and the number of states are calculated using the extended Beyer–Swinehart algorithm.

**Figure 4:**
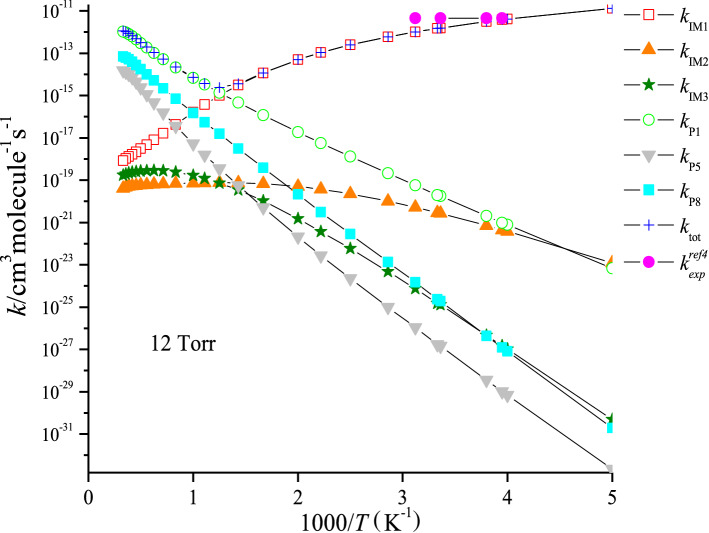
Temperature dependence of the total and individual rate coefficients for the CH_3_CFClO_2_• + ClO• reaction at 200–3,000 K at 12 Torr of N_2_. The experimental data are taken from Ref 5.

**Figure 5:**
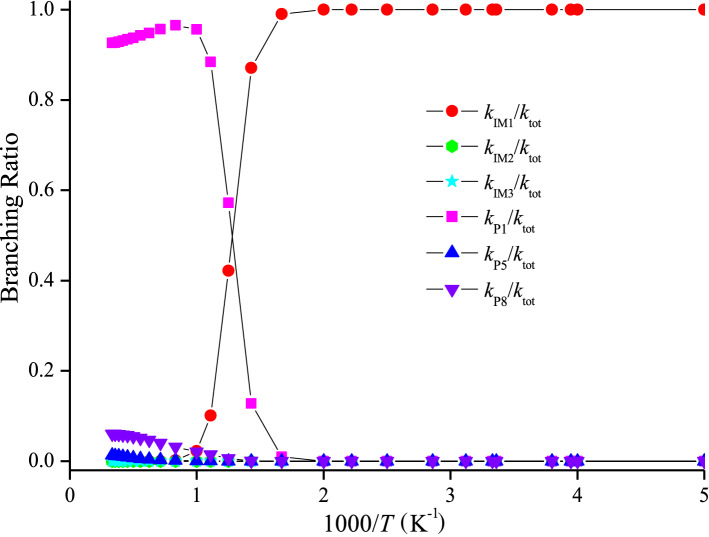
Branching ratios of the primary routes for the CH_3_CFClO_2_• + ClO• reaction at 12 Torr of N_2_.

The rate constants of IM1 (CH_3_CFClOOOCl), IM2 (CH_3_CFClOOClO) and IM3 (CH_3_CFClOClO_2_) collisional stabilization channels, and those for the P1 (CH3CFO + ClOOCl), P5 (CH3CFO + Cl_2_O_2_) and P8 (CH3CFO + ClOClO) channels (denoted as *k*_IM1_, *k*_IM2_, *k*_IM3_, *k*_P1_, *k*_P5_ and *k*_P8_) and the total rate coefficient (*k*_tot_ = *k*_IM1_ + *k*_IM2_ + *k*_IM3_ + *k*_P1_ + *k*_P5_ + *k*_P8_) at 200–3,000 K, 12 torr N_2_ are presented in Fig. 4. *k*_tot_ appears to reduce firstly and then increase with the temperature increasing. Meanwhile, *k*_tot_ was in accord with the experimental data (e.g. *k*_tot_ = 4.50 × 10^–12^ cm^3^ molecule^−1^ s^−1^ vs. *k*_tot(exp)_ = 3.84 × 10^–12^ cm^3^ molecule^-1^ s^-1^ at 253 K). The branching ratios are listed Fig. 5. The generation of IM1 (CH_3_CFClOOOCl) is dominated at 200–800 K, and with the production of the P1 (CH_3_CFO + ClOOCl) becoming predominant quickly with the rise of temperature. The P5 (CH_3_CFO + Cl_2_O_2_) or P8 (CH_3_CFO + ClOClO) product pathway generating from the IM2 (CH_3_CFClOOClO) or IM3 (CH_3_CFClOClO_2_) and the collisional stabilization of the IM2 (CH_3_CFClOOClO) and IM3 (CFCl_2_CH_2_OClO_2)_ almost don’t occur.

**Figure 6:**
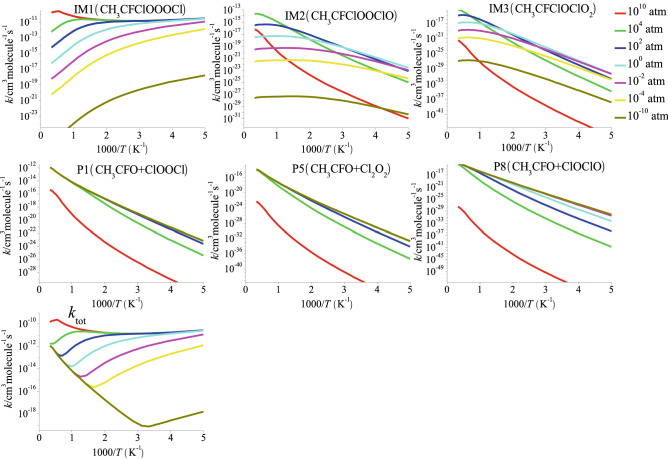
Forecasted rate coefficients for the total reaction and each individual product pathway of the CH_3_CFClO_2_• + ClO• reaction at 200–3,000 K and 10^–10^-10^10^ atm.

The rate constants for formation of individual products and total rate constants of the CH_3_CFClO_2_• + ClO• reaction at 200–3,000 K and 10^–10^–10^10^ atm are shown in Fig. 6. As seen from the figure, *k*_P1_, *k*_P5_, *k*_P8_, *k*_IM1_, *k*_IM2_ and *k*_IM3_ for the formation of IM1 (10^–10^–10^10^ atm), IM2 (10^–10^–10^2^ atm) and IM3 (10^–10^–10^2^ atm) by collisional deactivation is strongly pressure dependent with a pattern opposite to that of the decomposition processes and IM2 (10^4^–10^10^ atm) and IM3 (10^4^–10^10^ atm) by collisional deactivation because of the competitive effect of the stabilization vs decomposition as alluded to above. *k*_IM1_, *k*_IM2_ and *k*_IM3_ become smaller and less competitive at lower pressure; at pressure over 1 atm, *k*_IM1_ is approaching the high pressure limit at *T* ≤ 1,000 K. In addition, *k*_IM1_ displays negative dependence on temperature at 200–3,000 K owing to the reduction of collision inactivation rate, except at high-pressure limit pressure. The rate constants *k*_P1_, *k*_P5_ and *k*_P8_ for the dissociation reactions display positive dependence on temperature and negative pressure dependent. At low temperatures and high pressures, *k*_P1_ become insignificantly small. *k*_tot_ reflects positive pressure dependent. The high-pressure limit rate constants monotone increase firstly and then reduce monotonously with the temperatures increase, with a model contrary to the collisionless limit pressure, which may due to competition between addition and decomposition reaction.

**Figure 7:**
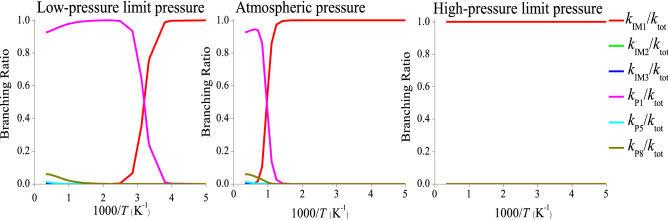
Forecasted branching ratios for the CH_3_CFClO_2_• with ClO• reaction at low-pressure limit pressure, atmospheric pressure and high-pressure limit pressure.

The branching ratios of the individual product pathways of the CH_3_CFClO_2_• + ClO• reaction at low-pressure limit (10^–10^ atm), atmospheric pressure (1 atm) and high-pressure limit pressure (10^10^ atm) are presented in Fig. 7. Six product channels dominant noticeably—the competitive deactivation and decomposition producing IM1, IM2, IM3, P1, P5 and P8, respectively. At high-pressure limit pressure, the formation of the stabilization product, IM1 (CH_3_CFClOOOCl) dominants the reaction at 200–3,000 K. At low-pressure limit and atmospheric pressure, the production of P1 (CH_3_CFO + ClOOCl) dominates the reaction at T ≥ 300 K and T ≥ 1,000 K, respectively; conversely at low temperatures, the collision inactivation of IM1 (CH_3_CFClOOOCl) dominants the reaction.

The three-parameter Arrhenius equations for the rate constants of generation of IM1 (CH_3_CFClOOOCl) (*k*_IM1_) and P1 (CH_3_CFO + ClOOCl) (*k*_P1_) at low-pressure limit, 1 atm and high-pressure limit N_2_ can be represented by:

$$ k_{{\rm{IM1}}}^{0} \left( {\rm{CH}_{\rm{3}} \rm{CFClOOOCl}} \right)/\left( {\rm{cm}^{\rm{3}} \rm{molecule}^{{\rm{ - 1}}} \rm{s}^{{\rm{ - 1}}} } \right) = {\text{ 6}}.{\text{12}} \times {\text{1}}0^{{ - {\text{2}}0}} T^{{ - 0.{\text{95}}}} {\text{exp}}\left( {{\text{1653}}.{\text{76}}/T} \right)~({\text{2}}00 \le T \le {\text{3}}000{\text{ K}}) $$

$$ k_{{\rm{P1}}}^{0} \left( {\rm{CH}_{\rm{3}} \rm{CFO + ClOOCl}} \right)/\left( {\rm{cm}^{\rm{3}} \rm{molecule}^{{\rm{ - 1}}} \rm{s}^{{\rm{ - 1}}} } \right) = {\text{ 9}}.{\text{2}}0 \times {\text{1}}0^{{ - {\text{23}}}} T^{{0.{\text{3}}0}} {\text{exp}}\left( { - {\text{6665}}.{\text{7}}/T} \right)~~~~~~({\text{2}}00 \le T \le {\text{3}}000{\text{ K}}) $$

$$  k_{{\rm{IM1}}}^{{}} \left( {\rm{CH}_{\rm{3}} \rm{CFClOOOCl}} \right)/\left( {\rm{cm}^{\rm{3}} \rm{molecule}^{{\rm{ - 1}}} \rm{s}^{{\rm{ - 1}}} } \right) = {\text{ 1}}.{\text{92}} \times {\text{1}}0^{{ - {\text{9}}}} T^{{ - {\text{1}}.{\text{22}}}} {\text{exp}}\left( {{\text{436}}.{\text{86}}/T} \right)~~~~~~~({\text{2}}00 \le T \le {\text{3}}000{\text{ K}})  $$

$$  k_{{\rm{P1}}}^{{}} \left( {\rm{CH}_{\rm{3}} \rm{CFO + ClOOCl}} \right)/\left( {\rm{cm}^{\rm{3}} \rm{molecule}^{{\rm{ - 1}}} \rm{s}^{{\rm{ - 1}}} } \right) = {\text{ 4}}.{\text{19}} \times {\text{1}}0^{{ - {\text{13}}}} T^{{0.{\text{39}}}} {\text{exp}}\left( { - {\text{6454}}.{\text{79}}/T} \right)~~~~~({\text{2}}00 \le T \le {\text{3}}000{\text{ K}}) $$

$$  \begin{gathered}   k_{{\rm{IM1}}}^{\infty } \left( {\rm{CH}_{\rm{3}} \rm{CFClOOOCl}} \right)/\left( {\rm{cm}^{\rm{3}} \rm{molecule}^{{\rm{ - 1}}} \rm{s}^{{\rm{ - 1}}} } \right) = {\text{ 2}}.{\text{86}} \times {\text{1}}0^{{ - {\text{16}}}} T^{{{\text{1}}.{\text{84}}}} {\text{exp}}\left( { - {\text{425}}.{\text{41}}/T} \right)~~~~~~({\text{2}}00 \le T \le {\text{18}}00{\text{ K}}) \hfill \\    = {\text{ 3}}.{\text{38}} \times {\text{1}}0^{{ - {\text{6}}}} T^{{ - {\text{1}}.{\text{21}}}} {\text{exp}}\left( { - {\text{811}}.{\text{52}}/T} \right)~~~~~({\text{18}}00 < T \le {\text{3}}000{\text{ K}}) \hfill \\  \end{gathered}   $$

 

$$ k_{{\rm{P1}}}^{\infty } \left( {\rm{CH}_{\rm{3}} \rm{CFO + ClOOCl}} \right)/\left( {\rm{cm}^{\rm{3}} \rm{molecule}^{{\rm{ - 1}}} \rm{s}^{{\rm{ - 1}}} } \right) = {\text{ 3}}.{\text{15}} \times {\text{1}}0^{{ - {\text{13}}}} T^{{ - 0.{\text{42}}}} {\text{exp}}\left( { - {\text{9664}}.{\text{7}}/T} \right)~~~~~({\text{2}}00 \le T \le {\text{3}}000{\text{ K}}) $$.

### Atmospheric lifetimes of CH_3_CFClO_2_

The atmospheric lifetime of CH_3_CFClO_2_• can be deduced by means of the following formula:$\tau = \frac{{1}}{{k\left[ {\rm{ClO}} \right]}}$. The calculated average daytime atmospheric concentrations of chlorine monoxide radical (ClO•) are 1 × 10^7^ molecules per cm^3^ ^26^, and *k*_ClO_ = 8.49 × 10^–12^ molecules per cm^3^ at 298 K 760 Torr was considered. The atmospheric lifetime of CH_3_CFClO_2_• is approximately 3.27 h, which suggests that ClO-initiated reaction of CH_3_CFClO_2_• plays an important role in some special areas and the marine boundary layer.

### Vertical excitation energy of IM1 (CH_3_CFClOOOCl), IM2 (CH_3_CFClOOClO) and IM3 (CH_3_CFClOClO_2_)

The photo-oxidation of compounds containing chlorine is significant for Cl atmospheric chemistry. As source of Cl, the photolysis might influence the stratosphere and troposphere. In order to get new insights of photolytic stability of the chlorinated compounds, the vertical excitation energy (*T*_*V*_) of the first five excited states for IM1 (CH_3_CFClOOOCl), IM2 (CH_3_CFClOOClO) and IM3 (CH_3_CFClOClO_2_) were calculated by employing TDDFT method based on the B3LYP/6–311 +  + G(d,p) optimized geometries, and the calculation results including wavelength (λ), excitation energy (*T*_*V*_) and oscillator strength (*f*) are listed in Table 2. Compounds will be considered to photolyze if *T*_*V*_ value is smaller than 4.13 eV or wavelength is longer than 300 nm. From Table 2 it is seen that the *T*_*V*_ (wavelength) value of the first two excited states of IM1 (CH_3_CFClOOOCl) are 2.95 eV (420.0 nm), 3.96 eV (313.4 nm) and the oscillator strength are 0.0001, 0.0001, and the second excited states of IM2 (CH_3_CFClOOClO) is 3.65 eV (339.70 nm) and the oscillator strength is 0.0007, indicating the possibility of photolysis of IM1 (CH_3_CFClOOOCl) and IM2 (CH3CFClOOClO) under the sunlight. As for IM3 (CH_3_CFClOClO_2_), *T*_*V*_ value and oscillator strength the first three excited states take values of 3.28 eV (378.2 nm), 3.78 eV (327.7 nm) 4.10 eV (302.6 nm) and 0.0029, 0.0742, 0.0007, respectively, implying the photolysis is feasible under the sunlight, which might be one source of Cl-containing species in the atmosphere.

**Table 2:** The excitation energy *T*_*V*_ (in eV), oscillator strength *f* (in atomic units) and wavelength *λ* (in nm) of the first five excited states of IM1 (CH_3_CFClOOOCl), IM2 (CH_3_CFClOOClO) and IM3 (CH_3_CFClOClO_2_) at the TD-B3LYP level of theory.

**Excited states**	**IM1 (CH**_**3**_**CFClOOOCl)**			**IM2 (CH**_**3**_**CFClOOClO)**			**IM3 (CH**_**3**_**CFClOClO**_**2**_**)**		
	***T***_***V***_	***f***	***λ***	***T***_***V***_	***f***	***λ***	***T***_***V***_	***f***	***λ***
1	2.95	0.0001	420.0	1.87	0.0000	663.25	3.28	0.0029	378.2
2	3.96	0.0001	313.4	3.65	0.0007	339.70	3.78	0.0742	327.7
3	4.42	0.0016	280.1	4.56	0.2238	271.83	4.10	0.0007	302.6
4	4.95	0.0381	250.4	4.90	0.0002	252.96	4.54	0.0005	272.7
5	5.55	0.2008	223.2	5.44	0.0020	228.05	4.72	0.0044	262.8

## Conclusions

The reaction mechanisms, kinetics, and products distribution for the CH_3_CFClO_2_• + ClO• reaction in atmosphere were investigated by using the CCSD(T)//B3LYP method. Addition–elimination, direct H-abstraction and S_N_2 displacement mechanisms are located on the singlet PES, and direct H-abstraction and S_N_2 displacement mechanisms are located on the triplet PES. The result suggests that major product is P1 (CH_3_CFO + ClOOCl) on the singlet PES produced by the addition–elimination reaction, which proceeds the addition of the O in ClO to the terminal-O atom in CH_3_CFClO_2_• and then the ClOOCl-elimination forming the products. Owing to the higher barrier heights, other products contribute less to the title reaction. The rate constants and branch ratio of products are estimated by means of RRKM theory at extensive temperature and pressure range. The rate constants at 200–3,000 K show stronger temperature dependence. The stabilization of the adduct IM1 (CH_3_CFClOOOCl) is dominant at 200–800 K, while the generation of P1 (CH_3_CFO + ClOOCl) is the primary channel at high temperature. The lifetime of CH_3_CFClO_2_• in the presence of ClO• was predicted to 3.27 h. IM1 (CH_3_CFClOOOCl), IM2 (CH_3_CFClOOClO) and IM3 (CH_3_CFClOClO_2_) will photolyze under the sunlight.

## Supplementary information

Supplementary information
