# Supplementary material for: Theoretical investigations on mechanisms and kinetics of the CH3CFClO2· with ClO· reaction in the atmosphere
Source: Sci Rep. 2020 Jul 6;10:11078. doi: 10.1038/s41598-020-68049-4 (PMC7338532; doi:10.1038/s41598-020-68049-4)
Supplement: Supplementary file 1 — Supplementary information [file 41598_2020_68049_MOESM1_ESM.docx]

**Theoretical Investigations on Mechanisms and kinetics of the CH_3_CFClO_2_• with ClO• Reaction in the Atmosphere**

Yunju Zhang,^^[[1]](#footnote-1)^*^ Bing He,^2^ Yuxi Sun,^1^

*^1^Key Laboratory of Photoinduced Functional Materials, Mianyang Normal University, Mianyang 621000, PR China*

*^2^College of Chemistry and Life Science, Institute of functional molecules, Chengdu Normal University, Chengdu, Sichuan 611130, PR China*

**Table S1.** The harmonic vibrational frequencies (imaginary frequency is suffixed with *i*) and the moment of inertia (*I*_a_, I_b_ and I_c_) of all the species in the CH_3_CFClO_2_• with ClO• reaction.

| Species | Frequencies (cm^-1^) | *I*_a_, *I*_b_, *I*_c_ (amu bohr^2^) |
| --- | --- | --- |
| CH_3_CFClO_2_• | 114, 228, 245, 302, 371, 408, 422, 610, 666, 803, 925, 1098, 1138, 1169, 1223, 1420, 1477, 1479, 3061, 3140, 3154 | 537.69344, 648.11070, 837.58633 |
| ClO• | 809(*854*) | 0.00000, 102.40242, 102.40242 |
| IM1 | 40, 62, 142, 181, 257, 299, 316, 340, 389, 424, 469, 540, 567, 636, 744, 858, 951, 1006, 1127, 1155, 1203, 1418, 1478, 1483, 3066, 3143, 3156 | 686.21717, 2556.05891, 2698.74016 |
| IM2 | 39, 81, 95, 180, 212, 224, 291, 325, 368, 405, 415, 513, 606, 668, 872, 878, 916, 933, 1130, 1154, 1201, 1415, 1476, 1480, 3062, 3141, 3155 | 695.12130, 2242.46652, 2307.07012 |
| IM3 | 27, 63, 113, 208, 221, 239, 312, 325, 391, 421, 451, 487, 627, 655, 842, 967, 979, 1098, 1127, 1139, 1209, 1402, 1470, 1479, 3060, 3140, 3158 | 765.03064, 1832.81288, 2131.26465 |
| TS1 | 147*i*, 45, 55, 113, 191, 244, 278, 309, 381, 416, 459, 507, 552, 741, 779, 854, 929, 1034, 1128, 1160, 1188, 1415, 1476, 1481, 3064, 3147, 3151 | 512.08072, 3062.48043, 3113.18335 |
| TS2 | 157*i*, 36, 40, 120, 136, 189, 243, 278, 312, 399, 440, 442, 576, 593, 795, 880, 984, 1043, 1061, 1119, 1277, 1390, 1470, 1477, 3056, 3136, 3161 | 741.06814, 2699.00661, 2855.36686 |
| TS3 | 298*i*, 32, 72, 113, 145, 196, 208, 233, 258, 346, 412, 434, 545, 564, 631, 824, 1013, 1025, 1186, 1342, 1392, 1434, 1465, 1487, 3043, 3126, 3174 | 751.52130, 2846.87248, 3158.38507 |
| TS4 | 451*i*, 36, 46, 98, 136, 156, 233, 254, 295, 356, 365, 409, 464, 500, 559, 588, 946, 1031, 1089, 1394, 1408, 1461, 1474, 1507, 3050, 3124, 3171 | 506.18506, 3680.51221, 3794.71930 |
| TS5 | 927*i*, 31, 47, 109, 123, 235, 277, 319, 338, 398, 430, 480, 559, 641, 653, 738, 872, 1020, 1054, 1130, 1258, 1285, 1359, 1396, 1532, 3041, 3188 | 793.69304, 3009.91959, 3343.28941 |
| TS6 | 182*i*, 34, 64, 139, 153, 158, 197, 223, 237, 347, 401, 422, 459, 670, 709, 992, 999, 1014, 1093, 1202, 1324, 1382, 1421, 1470, 2986, 3084, 3197 | 1083.92936, 1823.62583, 2336.08636 |
| TS7 | 242*i*, 45, 70, 147, 171, 206, 214, 266, 298, 348, 425, 438, 544, 590, 791, 806, 1000, 1030, 1036, 1121, 1348, 1395, 1458, 1477, 3052, 3132, 3169 | 795.88504, 1858.66416, 2034.11843 |
| TS8 | 469*i*, 28, 87, 150, 184, 195, 226, 257, 265, 312, 363, 417, 462, 511, 577, 899, 964, 1025, 1078, 1118, 1378, 1452, 1461, 1528, 3051, 3133, 3149 | 795.11856, 1816.36200, 2000.70373 |
| TS9 | 320*i*, 42, 103, 126, 153, 165, 223, 274, 311, 360, 376, 410, 456, 469, 641, 769, 956, 1029, 1155, 1250, 1397, 1424, 1450, 1470, 3025, 3117, 3175 | 1066.14634, 1532.67709, 1990.10554 |
| TS10 | 319*i*, 32, 73, 161, 181, 191, 223, 255, 288, 321, 422, 466, 543, 590, 805, 935, 999, 1029, 1112, 1162, 1392, 1414, 1461, 1477, 3043, 3123, 3166 | 834.18465, 1764.19675, 2167.46309 |
| TS11 | 433*i*, 17, 62, 120, 170, 181, 201, 229, 287, 322, 367, 435, 462, 499, 590, 911, 962, 1020, 1087, 1106, 1387, 1452, 1465, 1590, 3047, 3126, 3157 | 790.61612, 1997.40345, 2274.88125 |
| TS12 | 207*i*, 39, 59, 63, 85, 93, 115, 164, 203, 241, 244, 344, 377, 451, 681, 733, 987, 1012, 1227, 1290, 1398, 1456, 1459, 1471, 3032, 3104, 3159 | 673.33037, 2484.59153, 2723.94814 |
| TS13 | 551*i*, 41, 81, 87, 118, 130, 160, 200, 237, 253, 342, 360, 423, 494, 660, 749, 971, 1026, 1179, 1329, 1411, 1423, 1455, 1483, 3040, 3105, 3141 | 649.78049, 2546.29787, 2724.93659 |
| h-TS1 | 919*i*, 25, 46, 58, 120, 165, 244, 302, 328, 363, 431, 501, 548, 636, 669, 742, 873, 968, 1034, 1149, 1201, 1234, 1279, 1420, 1455, 3077, 3173 | 882.55776, 2848.42748, 3254.07628 |
| T-h-TS1 | 1645*i*, 27, 40, 73, 112, 128, 240, 256, 362, 389, 408, 508, 609, 644, 664, 730, 808, 922, 979, 1110, 1150, 1183, 1242, 1347, 1438, 3117, 3214 | 714.63443, 2991.23601, 3245.87817 |
| T-TS1 | 459*i*, 26, 42, 69, 99, 111, 137, 148, 179, 231, 299, 339, 368, 437, 643, 772, 979, 1004, 1187, 1282, 1402, 1421, 1457, 1470, 3024, 3093, 3149 | 645.57379, 2783.09396, 3027.26495 |
| T-TS2 | 792*i*, 36, 49, 51, 118, 181, 201, 254, 289, 328, 378, 424, 517, 541, 666, 754, 825, 952, 1086, 1158, 1214, 1405, 1478, 1482, 3062, 3142, 3151 | 482.07792, 3433.01648, 3510.62557 |
| T-TS3 | 505*i*, 25, 56, 99, 113, 150, 185, 273, 283, 316, 337, 416, 445, 542, 681, 767, 839, 911, 1105, 1145, 1157, 1408, 1475, 1483, 3068, 3147, 3164 | 810.18193, 2336.23008, 2622.46697 |
| CH_3_CFClOOCl | 55, 92, 219, 239, 277, 323, 363, 434, 452, 537, 630, 735, 812, 843, 940, 1130, 1154, 1197, 1418, 1480, 1482, 3066, 3145, 3152 | 494.01234, 1886.57134, 1947.04642 |
| cyc-CFClOCH_2_ | 310, 357, 414, 504, 605, 782, 899, 1036, 1098, 1137, 1170, 1384, 1517, 3115, 3221 | 270.20981, 501.29878, 645.73218 |
| CH_3_CFClClO | 41, 151, 235, 251, 277, 328, 363, 436, 520, 673, 751, 935, 1080, 1140, 1180, 1416, 1471, 1476, 3046, 3113, 3150 | 623.16119, 933.24643, 1185.15327 |
| CH_3_CFClOCl | 119, 187, 235, 299, 337, 398, 429, 544, 651, 721, 884, 937, 1118, 1167, 1177, 1411, 1474, 1481, 3063, 3143, 3158 | 571.01677, 1018.69900, 1238.34137 |
| CH_3_CFClClO | 41, 151, 235, 251, 277, 328, 363, 436,520, 673, 751, 935, 1080, 1140, 1180, 1416, 1471, 1476, 3046, 3113, 3150 | 623.16119, 933.24643,1185.15327 |
| CH_2_CFClO_2_ | 114, 165, 241, 286, 374, 403, 407, 562, 633, 657, 804, 909, 1094, 1163, 1253, 1434, 3174, 3299 | 523.54360, 637.27533, 816.68825 |
| CH_3_CFClO | 216, 236, 265, 342, 418, 460, 557, 803, 969, 1028, 1141, 1254, 1399, 1472, 1476, 3055, 3132, 3162 | 333.86871, 558.83049, 580.75767 |
| CH_3_CFCl_2_ | 258, 268, 295, 375, 391, 427, 579, 718, 918, 1106, 1122, 1161, 1418, 1477, 1478, 3059, 3135, 3152 | 476.17608, 723.23307, 847.54341 |
| CH_3_CClO | 132(*166*), 342(*348*), 428(*445*), 518(*518*), 597(*604*), 955(*956*), 1044(*1032*), 1113(*1108*), 1390(*1368*), 1465(*1415*), 1466(*1431*), 1891(*1818*), 3050(*2948*), 3120(*2990*), 3147(*3027*) | 178.10496, 374.52187, 541.47308 |
| CH_3_CFO | 127(*123*), 413(*420*), 569(*567*), 595(*598*), 815(*826*), 1000(*1000*), 1068(*1054*), 1180(*1188*), 1401(*1378*), 1469(*1437*), 1474(*1440*), 1913(*1870*), 3052(*2955*), 3114(*3004*), 3161(*3043*) | 164.71984, 187.14534, 340.70301 |
| ClOOCl | 124, 296, 406, 536, 587, 923 | 139.30927, 842.57752, 942.77093 |
| ClOClO | 90, 205, 314, 394, 637, 925 | 127.80636, 799.63378, 873.76522 |
| FOClO | 146, 262, 338, 417, 823, 941 | 102.17548, 509.82773, 555.77748 |
| FOOCl | 166, 339, 487, 571, 665, 1088 | 104.32757, 574.20102, 642.03784 |
| FClO_2_ | 289, 343, 498, 560, 1015, 1153 | 198.30305, 246.16412, 396.65650 |
| HOCl | 700(*724*), 1226(*1239*), 3777(*3609*) | 2.95969, 124.65718, 127.61688 |
| OClO | 412(*448*), 864(*946*), 986(*1110*) | 36.69613, 194.86668, 231.56281 |
| Cl_2_O_2_ | 212, 237, 394, 473, 954, 1103 | 202.29216, 561.51199, 716.14097 |
| HO_2_ | 1156(*1098*), 1421(*1392*), 3602(*3436*) | 2.89488, 53.58440, 56.47928 |
| O_3_ | 746(*705*), 1183(*1042*), 1245(*1110*) | 15.72270, 133.05757, 148.78027 |
| O_2_(^3^Σ) | 1633(*1580*) | 0.00000, 41.51716, 41.51716 |
| O_2_(^1^Δ_g_) | 1622 | 0.00000, 41.49877, 41.49877 |

Numbers in italics stand for experimental data from Ref.21.

**Table S2.** The Cartesian coordinates of all the species in the CH_3_CFClO_2_• + ClO• reaction.

| Species |  | Coordinates(Atom, X, Y, Z) |
| --- | --- | --- |
| IM1 | C  O  O  O  Cl  C  F  Cl  H  H  H | -1.20883 0.25329 0.19476  -0.36141 -0.03176 -0.95721  0.81088 -0.64365 -0.6111  1.805 0.50343 -0.31877  3.19073 -0.28641 0.26752  -1.10138 1.69196 0.62838  -0.88904 -0.58588 1.19968  -2.85402 -0.15988 -0.40168  -1.3243 2.34746 -0.21261  -1.80455 1.88391 1.43909  -0.08099 1.87461 0.96978 |
| IM2 | C  O  O  F  O  Cl  Cl  C  H  H  H | 1.23448 -0.2878 0.14723  0.52043 -1.10631 -0.75175  -0.66153 -0.53852 -1.23321  0.58765 -0.35303 1.35849  -2.19698 0.95698 0.67555  -2.07762 -0.44442 0.03769  1.25016 1.43663 -0.34452  2.6196 -0.87324 0.26277  2.53904 -1.92619 0.53862  3.14715 -0.7794 -0.68566  3.16293 -0.33553 1.03993 |
| IM3 | C  O  O  O  Cl  C  H  H  H  F  Cl | -1.20632 0.38045 0.20998  -0.03058 0.72505 -0.32073  2.45854 1.02514 -0.81688  2.19577 -0.65742 1.0021  1.7921 -0.20091 -0.34313  -2.18087 1.52897 -0.05463  -1.77443 2.43296 0.39971  -2.29898 1.67275 -1.12659  -3.14141 1.28001 0.39763  -1.114 0.14176 1.56501  -1.93215 -1.18642 -0.48849 |
| TS1 | C  O  O  O  Cl  C  F  Cl  H  H  H | -1.27461 0.11657 0.20406  -0.41599 -0.33341 -0.88061  0.85868 -0.04424 -0.6283  2.91499 0.91391 -0.41916  2.87605 -0.52857 0.23267  -1.11907 1.5825 0.5073  -0.96775 -0.63991 1.29018  -2.90841 -0.3255 -0.36236  -1.37288 2.17454 -0.37129  -1.78311 1.84572 1.33026  -0.08343 1.77355 0.79047 |
| TS2 | C  O  O  O  Cl  C  H  H  H  F  Cl | -1.37303 0.27084 0.19412  -0.11528 0.06518 -0.00637  1.80331 1.11712 -0.55553  3.05367 -0.58621 0.9243  2.36069 -0.26383 -0.36583  -1.97321 1.48263 -0.51161  -1.49172 2.37542 -0.10784  -1.78773 1.41385 -1.58148  -3.04373 1.51811 -0.31196  -1.73176 0.30867 1.53099  -2.15561 -1.17264 -0.41124 |
| TS3 | C  O  O  O  Cl  C  H  H  H  F  Cl | 0.43633 -0.43199 -0.74282  -0.15422 -1.51996 -0.39462  -1.86796 -0.4669 0.27616  -2.93005 -0.37577 0.91114  -4.5205 -0.51052 -0.13623  1.62117 0.05273 0.13856  2.47009 -0.58156 -0.0095  1.34825 0.0223 1.17272  1.86538 1.06168 -0.12081  0.66524 -0.23635 -2.0588  -0.94438 1.07813 -0.4296 |
| TS4 | C  O  O  O  Cl  C  H  H  H  F  Cl | 0.43633 -0.43199 -0.74282  -0.15422 -1.51996 -0.39462  -1.86796 -0.4669 0.27616  -2.93005 -0.37577 0.91114  -4.5205 -0.51052 -0.13623  1.62117 0.05273 0.13856  2.47009 -0.58156 -0.0095  1.34825 0.0223 1.17272  1.86538 1.06168 -0.12081  -0.73761 0.85197 -0.47651  0.73477 -0.17694 -2.45847 |
| TS5 | C  O  O  O  Cl  C  F  Cl  H  H  H | 1.52666 0.26715 0.18635  0.75157 0.92091 -0.64682  -1.84249 1.39226 -0.98222  -2.00793 0.88484 0.11788  -2.90803 -1.01744 0.18102  0.29168 -0.09043 0.94416  2.40808 1.05272 0.88263  2.48412 -1.0897 -0.52719  -0.03121 -1.12394 0.82317  0.24504 0.42071 1.90735  -0.79937 0.40574 0.51692 |
| TS6 | C  O  O  O  Cl  C  H  H  H  Cl  F | 1.00269 0.57314 0.10224  -1.10812 1.52156 -0.15938  -2.15224 0.77022 0.14199  -2.09353 -0.39143 -0.36966  -0.53786 -1.48195 0.84442  1.55671 0.63685 1.48158  1.02817 1.27064 2.16264  2.51193 1.05034 1.23361  1.73553 -0.31627 1.9338  1.1245 2.11446 -0.73865  1.47479 -0.38527 -0.72303 |
| TS7 | C  O  O  O  Cl  C  H  H  H  Cl  F | 1.31112 -0.37353 -0.11202  0.81995 -1.07692 -0.99064  -1.35209 -0.60055 -1.2001  -2.23933 1.01734 0.56234  -1.88799 -0.363 0.1628  2.617 -0.73588 0.54723  2.61479 -1.8064 0.75063  3.44074 -0.4855 -0.12443  2.71206 -0.1712 1.47427  -0.00257 -0.51617 1.53296  1.21415 0.96261 -0.2788 |
| TS8 | C  O  O  F  O  Cl  Cl  C  H  H  H | -1.031 -0.10588 0.27815  -0.47768 -0.72522 1.16661  1.75818 -0.36126 1.26481  0.00933 -0.3989 -1.0096  2.69093 0.70642 -0.84414  2.17168 -0.48499 -0.14606  -1.19449 1.69579 0.40072  -2.32326 -0.72676 -0.28414  -2.23475 -1.79309 -0.28694  -3.15265 -0.43932 0.32773  -2.48024 -0.37927 -1.28389 |
| TS9 | C  O  O  O  Cl  C  F  Cl  H  H  H | -0.00996 0.68516 0.13349  -1.08624 -1.09214 0.56106  -1.86487 -0.89813 1.48831  -1.69576 0.11825 -0.98788  -3.09054 0.71271 -0.32253  1.28863 -0.05246 0.17413  -0.50072 1.079 1.32787  -0.07385 1.85635 -1.1787  1.3914 -0.6877 1.029  2.02592 0.71753 0.26592  1.44947 -0.62633 -0.71452 |
| TS10 | C  O  O  O  Cl  Cl  F  C  H  H  H | -1.19923 -0.5999 -0.07885  -0.23631 -0.9014 -0.83544  2.37161 -0.92119 0.72271  1.50045 1.29423 -0.13133  1.84716 -0.11873 -0.40819  -1.13496 1.53998 0.22999  -1.06253 -0.99533 1.22343  -2.60495 -0.80061 -0.57908  -3.30428 -0.37347 0.13995  -2.72083 -0.32329 -1.54912  -2.78042 -1.8766 -0.67233 |
| TS11 | C  O  O  O  Cl  F  Cl  C  H  H  H | 1.01692 0.03708 -0.32342  0.08317 -0.21873 -1.04727  -2.90882 0.99178 -0.11919  -1.72245 -1.04577 0.8313  -2.21516 -0.2995 -0.35381  0.19229 -0.39076 1.30112  1.53617 1.72086 -0.04156  2.19762 -0.9465 -0.22275  1.86271 -1.93225 -0.46979  2.96769 -0.65025 -0.90402  2.58281 -0.94038 0.77549 |
| TS12 | C  O  O  O  Cl  C  F  Cl  H  H  H | 1.24633 0.11023 0.24484  1.00623 0.94591 -0.92572  0.59436 2.1496 -0.57645  -1.87371 -0.9831 -0.06107  -3.19083 0.08692 -0.0257  0.07795 0.05215 1.17039  2.32646 0.63657 0.88708  1.68534 -1.46832 -0.43761  0.20454 -0.64681 1.99163  -0.32073 1.03011 1.43074  -0.98925 -0.5021 0.4845 |
| TS13 | C  O  O  O  Cl  C  H  H  H  Cl  F | 0.14886 0.91299 -0.12079  1.63381 1.42618 0.69827  2.08625 2.50576 0.29326  -1.77552 1.08759 -0.68749  -3.03813 0.27166 0.02886  0.38508 -0.59425 -0.22679  0.21345 -1.06207 0.72009  -0.26502 -1.03879 -0.95112  1.38291 -0.79008 -0.55976  -0.69524 1.57543 1.2743  0.32179 1.64883 -1.23933 |
| TS14 | C  O  O  Cl  O  C  F  Cl  H  H  H | 0.34697 0.51115 -0.14686  -0.99808 1.92842 -0.59162  -1.07579 2.87349 0.18608  2.54051 -0.47919 0.05018  3.49326 0.8097 -0.08396  -0.60658 -0.64721 0.05081  0.74143 1.2263 0.92807  0.94455 0.86926 -1.7631  -0.06607 -1.56114 0.18301  -1.19991 -0.4991 0.92883  -1.22557 -0.77449 -0.81265 |
| T-TS1 | C  O  O  O  Cl  C  H  H  H  F  Cl | -0.009 0.78233 -0.18063  -1.676 1.68148 0.23621  -1.63047 2.90544 0.08938  2.08609 0.63947 -0.83936  3.26199 0.1275 0.20319  -0.47099 -0.6555 -0.06173  0.33484 -1.34286 -0.21363  -1.21564 -0.86583 -0.80075  -0.85582 -0.83754 0.91994  0.03868 1.34661 -1.40611  0.79792 1.52663 1.19506 |
| T-TS2 | C  O  O  O  Cl  C  H  H  H  F  Cl | 0.83021 1.24078 -0.42959  -0.21651 1.70411 0.3499  -1.68084 1.0984 -0.36798  -4.02412 -0.79015 -0.5953  -2.85356 -0.08558 0.17961  1.5946 0.06369 0.19767  0.98088 -0.80506 0.31396  1.95632 0.33691 1.1669  2.43771 -0.16607 -0.4198  0.43161 0.91682 -1.67805  1.97336 2.57361 -0.54945 |
| T-TS3 | C  O  O  O  Cl  C  F  Cl  H  H  H | 0.26375 0.10054 -1.11873  -1.0017 0.6272 -0.76402  -0.87863 2.465 -0.79313  -1.98817 -0.79391 -0.67387  -3.10752 -0.59177 0.53309  1.1251 -0.26394 0.09714  0.89865 1.03096 -1.86284  0.01819 -1.34879 -2.0866  0.64589 -1.0187 0.68502  2.04826 -0.66355 -0.26752  1.30808 0.58045 0.72834 |
| T-TS4 | C  O  O  O  Cl  C  F  Cl  H  H  H | 0.88991 -0.13377 -0.40643  -0.37601 0.03544 -0.96966  -0.54896 1.521 -1.29434  -0.68725 -2.06685 2.58491  -1.48879 -3.73581 2.63022  1.1994 -1.49905 0.15926  1.07831 0.81835 0.56117  1.95264 0.27543 -1.80624  1.05012 -2.26006 -0.60523  2.23392 -1.51745 0.50032  0.33003 -1.76069 1.277 |
| CH_3_CFClO_2_ | C  O  O  C  Cl  F  H  H  H | 0.0838 0.38077 0.06089  -1.12677 0.26935 -0.61034  -1.89401 -0.6698 -0.14076  0.81404 1.55673 -0.52955  1.01862 -1.12541 -0.08008  -0.16548 0.57731 1.35293  0.20184 2.44104 -0.40003  1.75484 1.68556 -0.0125  0.99529 1.38825 -1.58184 |
| cyc-CFClOCH_2_ | C  O  C  F  Cl  H  H | 1.65093 0.22177 0.25418  0.63231 0.80992 -0.41553  0.35525 -0.01951 0.83432  2.47016 1.06667 0.90459  2.49677 -1.09051 -0.53516  -0.13949 -0.96827 0.64905  0.09329 0.5721 1.70758 |
| CH_3_CFCl_2_ | C  Cl  C  H  H  H  Cl  F | -0.08732 0.33088 0.  0.13405 -0.68641 -1.47258  0.85551 1.5103 0.  1.88929 1.1689 0.  0.6702 2.11165 0.89103  0.6702 2.11165 -0.89103  0.13405 -0.68641 1.47258  -1.37718 0.76565 0. |
| CH_3_CClO | C  O  C  H  H  H  Cl | 0.23823 -0.47964 0.  1.36876 -0.83426 0.  -0.99254 -1.33272 0.  -1.61356 -1.07324 -0.8587  -0.70122 -2.38163 0.  -1.61356 -1.07324 0.8587  -0.14923 1.3023 0. |
| CH_3_CFO | C  O  F  C  H  H  H | 0.1388 -0.11589 0.  1.12615 -0.77067 0.  -1.08153 -0.72854 0.  0.0018 1.36955 0.  -0.79251 1.68083 0.68471  0.95102 1.82514 0.  -0.79251 1.68083 -0.68471 |
| CH_2_CFClO_2_ | C  O  O  C  F  Cl  H  H | 0.90838 -0.12605 0.01094  1.08321 1.19355 -0.54262  1.89851 1.94896 0.1679  -0.07772 -0.84439 -0.83892  0.50126 -0.00974 1.29036  2.49093 -1.00009 0.00187  0.14862 -0.83109 -1.90131  -0.37902 -1.80839 -0.43783 |
| CH_3_CFClClO | C  Cl  O  C  F  Cl  H  H  H | 0.18448 0.47012 0.07602  2.1458 0.55684 -0.23248  2.78059 -0.90491 -0.07468  -0.15115 1.4642 1.14886  -0.35736 0.82415 -1.11121  -0.15722 -1.20101 0.43853  0.19206 2.4564 0.84619  -1.23532 1.48841 1.2797  0.32555 1.18533 2.08718 |
| CH_3_CFClOCl | C  O  Cl  C  F  Cl  H  H  H | 0.9105 -0.05323 0.29417  -0.36626 -0.1215 0.8671  -1.65967 -0.88155 -0.00305  1.79378 0.40557 1.43814  0.96139 0.84834 -0.72917  1.44006 -1.6175 -0.42511  1.41391 1.35956 1.8064  2.80936 0.53868 1.06458  1.78495 -0.33194 2.23863 |
| CH_3_CFClO | C  O  C  H  H  H  F  Cl | 1.47676 0.21339 0.06797  0.78298 -0.88519 -0.00393  1.12559 1.35384 -0.86091  0.14845 1.73372 -0.5556  1.07914 0.99415 -1.88716  1.86857 2.14542 -0.76993  1.64885 0.68521 1.34207  3.16183 -0.55803 -0.41912 |
| CH_3_CFClOOCl | C  O  O  Cl  C  F  Cl  H  H  H | 0.93014 -0.54772 0.16242  -0.24176 0.09617 -0.24842  -1.38417 -0.80141 0.11884  -2.80046 0.08318 -0.21454  0.97183 -2.03716 -0.08491  1.16041 -0.29612 1.48807  2.18475 0.33721 -0.78485  0.74306 -2.24992 -1.12807  1.96814 -2.4049 0.15738  0.2424 -2.53008 0.55847 |
| ClOOCl | O  O  Cl  Cl | 0.53064 0.39377 -0.38709  1.67204 0.64344 -0.71739  3.08056 -0.05919 0.27356  -0.70488 1.50717 0.08908 |
| ClOClO | O  O  Cl  Cl | 2.25291 -0.8838 0.71113  1.03038 1.55758 0.05011  1.86282 0.08346 -0.40823  -0.77868 1.74697 0.14524 |
| FOOCl | O  O  Cl  F | 1.03966 0.12541 -0.03283  2.09089 0.68226 0.04143  3.68439 -0.38631 -0.03153  -0.14949 -0.00161 0.88906 |
| FOClO | O  O  Cl  F | -2.51616 1.20585 0.12176  -1.48001 -1.12879 0.7875  -1.91601 -0.10002 -0.33644  0.04636 -0.83678 1.39502 |
| FClO_2_ | O  F  O  Cl | -1.51091 -0.62283 -1.03044  -0.41399 -0.67516 1.32985  -2.24905 1.01992 0.66311  -1.93932 -0.37375 0.34321 |
| Cl_2_O_2_ | O  O  Cl  Cl | 0.29117 1.11435 -1.24875  0.29117 1.11435 1.24875  -0.3324 0.62256 0.  0.05849 -1.66911 0. |
| OClO | O  O  Cl | -1.68274 0.47267 0.58164  -4.08123 0.24692 -0.39703  -2.76002 -0.41583 -0.02726 |
| HOCl | O  Cl  H | 0.03553 1.09647 0.  0.03553 -0.59972 0.  -0.88832 1.42343 0. |
| ClO | O  Cl | 1.63756 0.12734 0.02486  3.02835 -0.19598 -0.05307 |
| HO_2_ | O  O  H | 0.243 1.58861 -0.1652  0.6068 0.53885 -0.78251  0.00183 -0.22065 -0.55046 |
| O_3_ | O  O  O | 0. -1.07923 -0.21417  0. 0. 0.4284  0. 1.07923 -0.21417 |
| O_2_(^3^∑) | O  O | -2.14776 2.02344 0.36382  -1.73883 3.1273 0.1033 |
| O_2_(^1^∆_g_) | O  O | -0.6995 1.60699 0.15578  -1.41404 1.62394 -0.84353 |

1. * Corresponding author. Email address: [zhangyj010@nenu.edu.cn](mailto:zhangyj010@nenu.edu.cn) Tel.: +86 816 2200064; Fax: +86 816 2200819 [↑](#footnote-ref-1)
